# Sex Differences of Acute Stroke Treatment and in Hospital Outcomes After Intravenous Thrombolysis in Patients With Ischemic Stroke

**DOI:** 10.3389/fneur.2020.545860

**Published:** 2020-10-08

**Authors:** Bin Cai, Sheng-de Li, Hang Li, Zhen-qian Liu, Bin Peng

**Affiliations:** ^1^Department of Neurology, Peking Union Medical College Hospital, Peking Union Medical College & Chinese Academy of Medical Sciences, Beijing, China; ^2^Cerebrovascular Diseases Hospital, Henan Provincial People's Hospital, Zhengzhou, China; ^3^Xuzhou Mineral Coal Mining Group General Hospital, Xuzhou, China

**Keywords:** sex, ischemic stroke, intravenous thrombolysis, complications, in hospital outcomes

## Abstract

**Background:** Many studies have suggested that the clinical features of male patients with ischemic stroke are different from those of female patients, but related data on Chinese patients are scarce. Therefore, this study aimed to identify the differences in treatment delays, complications related to intravenous thrombolysis, and prognosis between male and female patients with ischemic stroke in China.

**Methods:** The data of patients with ischemic stroke who received intravenous thrombolysis were retrospectively analyzed. The data were obtained from the China Hospital Stroke Registry from January 2017 to April 2019. The general clinical characteristics, onset-to-door time, door-to-needle time, complications related to thrombolysis, National Institute of Health Stroke Scale (NIHSS) scores, and in-hospital mortality were compared between male and female patients to identify any sex differences in these factors. A multi-factorial analysis was conducted to explore whether sex is associated with in-hospital mortality and complications of intracerebral hemorrhage after thrombolysis.

**Results:** A total of 26,475 patients with ischemic stroke who received intravenous thrombolysis were involved in the study. The data were collected from 902 hospitals in 29 provinces, autonomous regions, and municipalities in China. The door-to-needle time was longer in female than in male patients (49 [35, 67] vs. 48 [35, 65], *P* = 0.008). Furthermore, the frequencies of intracerebral hemorrhage (4.1 vs. 3.2%, *P* < 0.001) and in-hospital mortality (2.55 vs. 1.83%, *P* < 0.001) were higher in female vs. male patients. However, sex was not associated with intracerebral hemorrhage and in-hospital mortality according to the adjusted multi-factorial analysis. In addition, improvement in NIHSS scores was greater in female patients than in male patients [−3 (−6, −1) vs. −3 (−5, −1), *P* = 0.036].

**Conclusions:** After adjusting for other predictors sex was not associated with intracerebral hemorrhage after thrombolysis or in-hospital mortality. Further study is warranted to evaluate the long-term outcomes in the different sexes.

## Introduction

Approximately 25,700,000 patients suffer stroke globally. Moreover, about 6,500,000 patients die from stroke annually, with 75.2% of the deaths occurring in developing countries (1). In China, the incidence of stroke is estimated to be 1,114.8/100,000, and the prevalence of new-onset stroke is about 2,400,000 per year, which leads to almost 1,100,000 deaths per year (2). The burden of stroke is predicted to become increasingly heavy in China owing to aging population increases and changes in lifestyle, and the prevalence of stroke will increase by 6.7% annually (3). Intravenous thrombolysis has been established as one of the most effective treatments in patients with acute ischemic stroke within the time window, which leads to improvement in prognosis and reduction in mortality rates in these patients (4).

According to a number of studies, significant differences exist between male and female patients with ischemic stroke with regard to etiology, risk factors, clinical manifestations, delays in pre- and in-hospital treatments, thrombolysis implementation, and complications and prognosis related to thrombolytic treatment (5–7). Investigations on these differences are critical for improving individual management between male and female patients with ischemic stroke, promoting the implementation of thrombolytic treatment, improving functional outcomes, and reducing mortality. However, large-sample studies on the abovementioned outcomes are scarce in Chinese patients with acute ischemic stroke. Thus, the current study aimed to analyze the differences in pre- and in-hospital treatment delays, complications related to intravenous thrombolysis, and prognosis between male and female patients with acute ischemic stroke based on integrated data from the China Hospital Stroke Registry. The obtained findings are expected to improve the emergency treatment level in male and female patients with ischemic stroke in China.

## Methods

### Source of Data

All study data were derived from the domestic big data platform–China Hospital Stroke Registry–from January 2017 to April 2019. As an important national stroke prevention project, the database was founded and managed by the office of stroke prevention project, the National Health Commission in China. The details on the management and operation of the database are described in the official website (http://www.chinasdc.cn). In brief, the database is a national cross-sectional survey that covers all primary and comprehensive stroke centers in China. Information on emergency treatments including intravenous thrombolysis were recorded by each center via the online platform, and all data were entered and uploaded to the national stroke database by experienced physicians and nurses. Trained data administrators were in charge of discriminating incomplete or inaccurate data based on standardized quality control and data cleaning processes.

This retrospective study was exempt from the requirement for informed consent, as neither individual information nor biological samples were involved. The study protocol was approved by the ethics committee of Peking Union Medical College Hospital of the Chinese Academy of Medical Sciences (review number: S-K988).

### Study Population and Data Collection

Patients with ischemic stroke aged 18–90 years who received intravenous thrombolytic treatment were enrolled in the study. Incomplete and obviously inaccurate data were excluded.

Demographic and clinical data, including sex; age; time of onset, hospitalization, and thrombolysis; National Institute of Health Stroke Scale (NIHSS) scores; Trial of ORG 10172 in Acute Stroke Treatment (TOAST) classification; transfer approach; thrombolytic agent; hospital level; complications; NIHSS scores after thrombolysis; and in-hospital all-cause mortality, were extracted from the database for analysis. Complications of intracerebral hemorrhage were determined using a brain computed tomography scan, and both symptomatic and asymptomatic hemorrhages were included. The onset-to-door time (ODT) and door-to-needle time (DNT) were applied to evaluate delays in pre- and in-hospital treatments. The responses to thrombolysis were assessed using the value differences among the NIHSS scores before and immediately, 24 h, and 7 days after thrombolysis.

### Statistical Analysis

Continuous variables are shown as mean ± standard deviation and median (interquartile range) in normal and non-normal distributions, respectively, and categorical variables are shown as frequency (proportion). Differences in normally and non-normally distributed continuous variables were identified using the Student's *t*-test and Mann-Whitney U test, respectively, while the chi-squared test was used for categorical variables. The demographic and clinical characteristics between male and female patients with ischemic stroke, including age, baseline NIHSS scores, ODT, DNT, TOAST classification, transfer approach, level of hospital admission, and thrombolytic agents, were compared. In-hospital mortality and complications related to thrombolysis were also compared between male and female patients. Based on the improvement in NIHSS scores from before to after thrombolysis, the differences in responses to thrombolysis were evaluated between male and female patients. Uni- and multi-factorial logistic regression analyses were applied to calculate the odds ratio (OR) and relative 95% confidence interval (CI) to assess the association between sex and in-hospital mortality. Potential variables that may be related to outcomes and those with a *P*-value <0.1 in the uni-factorial regression analysis were further validated by multi-factorial regression analysis to explore the relationship between sex and in-hospital mortality as well as intracerebral hemorrhage. A *P*-value of <0.05 was considered to reflect statistical significance, and all data were analyzed using IBM SPSS version 25.0 (SPSS Inc., Chicago, IL, USA).

## Results

### General Clinical Data

A total of 26,475 patients with acute ischemic stroke who received intravenous thrombolysis at 902 hospitals in 29 provinces, autonomous regions, and municipalities in China were included in the study. The median age of all participants was 66 (57, 74) years. There were 16,914 (63.9%) male and 9,561 (36.1) female patients. The age of onset, baseline NIHSS score, and rates of cardioembolism and transfer by ambulance were higher in female than in male patients (all *P*-values <0.05) (Table 1).

**Table 1:** Characteristics of participants by genders (*n* = 26475).

**Characteristics**	**All (*n* = 26,475)**	**Male (*n* = 16,914)**	**Female (*n* = 9,561)**	***P* value**
Age, years, M(Q_R_)	66 (57, 74)	65 (56, 72)	70 (62, 77)	<0.001
Age, *n* (%)				<0.001
≤ 45	1,261 (4.8%)	1,006 (5.9%)	255 (2.7%)	
46–65	11,152 (42.1%)	7,914 (46.8%)	3,238 (33.9%)	
66–80	11,203 (42.3%)	6,648 (39.3%)	4,555 (47.6%)	
≥81	2,859 (10.8%)	1,346 (8.0%)	1,513 (15.8%)	
NIHSS, M(Q_R_)	6 (3, 12)	6 (3, 11)	7 (3, 13)	<0.001
TOAST, *n* (%)				<0.001
LAA	13,694 (51.7%)	9,107 (53.8%)	4,587 (48.0%)	
CE	3,447 (13.0%)	1,705 (10.1%)	1,742 (18.2%)	
SVO	7,939 (30.0%)	5,204 (30.8%)	2,735 (28.6%)	
OD	267 (1.0%)	167 (1.0%)	100 (1.0%)	
UD	1,128 (4.3%)	731 (4.3%)	397 (4.2%)	
Arrival mode, *n*(%)				<0.001
Ambulance	10,161 (38.4%)	6,285 (37.2%)	3,876 (40.5%)	
Other	16,314 (61.6%)	10,629 (62.8%)	5,685 (59.5%)	
Treatment, *n* (%)				0.247
rt-PA	21,585 (81.5%)	13,748 (81.3%)	7,837 (82.0%)	
Urokinase	4,614 (17.4%)	2,980 (17.6%)	1,634 (17.1%)	
Unknown	276 (1.0%)	186 (1.1%)	90 (0.9%)	
Hospital levels, *n* (%)				0.043
Secondary	6,233 (23.5%)	3,915 (23.1%)	2,318 (24.2%)	
Tertiary	20,242 (76.5%)	12,999 (76.9%)	7,243 (75.8%)	

*NIHSS, National Institute of Health stroke scale; TOAST, trial of org 10,172 in acute stroke treatment; LAA, large artery atherosclerosis; CE, cardioembolism; SVO, small vessel occlusion; OD, other determined; UD, undetermined*.

### Pre- and In-Hospital Delays

The arrival rate in hospital within 3 h from onset was higher in female than in male patients (*P* = 0.009), but the ODT was not statistically different between male and female patients (*P* = 0.058). However, the DNT was longer and the rate of thrombolysis within 1 h from admission was lower in female patients than in male patients (*P* = 0.008 and 0.011, respectively) (Table 2).

**Table 2:** Pre- and in-hospital delay by genders.

**Index**	**All (*n* = 26,475)**	**Male (*n* = 16,914)**	**Female (*n* = 9,561)**	***P-*value**
ODT, min, M (Q_R_)	115 (66, 166)	115 (66, 169)	114 (66, 163)	0.058
ODT, *n* (%)				0.009
≤ 180 min	21,766 (82.2%)	13,827 (81.7%)	7,939 (83.0%)	
≥181 min	4,709 (17.8%)	3,087 (18.3%)	1,622 (17.0%)	
DNT, min, M (Q_R_)	48 (35, 65)	48 (35, 65)	49 (35, 67)	0.008
DNT, *n* (%)				0.011
≤ 60 min	18,833 (71.1%)	12,122 (71.7%)	6,711 (70.2%)	
≥61 min	7,642 (28.9%)	4,792 (28.3%)	2,850 (29.8%)	

*ODT, onset to door time; DNT, door to needle time*.

### In-Hospital Outcomes

A total of 554 (2.09%) cases of in-hospital all-cause mortality occurred, including 310 (1.83%) male and 244 (2.55%) female patients. The in-hospital mortality rate was higher in female than in male patients according to the uni-factorial logistic regression (OR: 1.403, 95% CI: 1.184–1.662, *P* < 0.001). Nonetheless, the association between sex and in-hospital mortality diminished after adjusting for age, baseline NIHSS score, DNT, TOAST classification, hospital level, and transfer approach (OR: 0.907, 95% CI: 0.757–1.086, *P* = 0.288).

Among the enrolled patients, NIHSS scores recorded immediately, 24 h, and 7 days after thrombolysis could be obtained in 25,314, 24,065, and 22,065 patients, respectively. The NIHSS scores immediately [5 (2, 8) vs. 4 (2, 9), *P* < 0.001], 24 h [4 (1, 10) vs. 3 (1, 11), *P* < 0.001], and 7 days [2 (0, 6) vs. 2 (0, 5), *P* < 0.001] after thrombolysis were all higher in female patients than in male patients.

The differences in NIHSS scores among before and immediately, 24 h, and 7 days after thrombolysis were also compared between male and female patients through Mann-Whitney U test. At 7 days after thrombolysis, female patients experienced a greater reduction in NIHSS scores than did male patients [−3 (−6, −1) vs. −3 (−5, −1), *P* = 0.036], indicating better functional improvement in female patients than in male patients at 7 days after thrombolysis. Besides, the delta NIHSS score between male and female patients was still significantly different at 7 days after adjusting for baseline variables including age, TOAST classification, treatment, arrival mode and hospital levels (*P* < 0.001). The differences among before and immediately and 24 h after thrombolysis were not significantly different between female and male patients [immediately after thrombolysis: −1 (−3, 0) vs. −1 (−3,0), *P* = 0.094; 24 h after thrombolysis: −2 (−4, 0) vs. −2 (−4, 0), *P* = 0.237].

### Complications Related to Thrombolysis

Table 3 shows the complications related to thrombolysis. The incidence of intracerebral hemorrhage and gingival bleeding was higher in female patients than in male patients (*P* < 0.05). Uni-factorial regression analysis showed that the risk of intracerebral hemorrhage after thrombolysis was higher in female than in male patients (OR: 1.287, 95% CI: 1.128–1.468, *P* < 0.001). However, sex was no longer associated with intracerebral hemorrhage after adjusting for age, baseline NIHSS score, DNT, TOAST classification, and transfer approach (OR: 0.982, 95% CI: 0.856–1.126, *P* = 0.790) (Table 4).

**Table 3:** Complications associated with thrombolysis by genders.

**Complication**	**All**	**Male**	**Female**	***P-*value**
Intracranial hemorrhage, *n* (%)	944 (3.6%)	549 (3.2%)	395 (4.1%)	<0.001
gastrointestinal hemorrhage, *n* (%)	195 (0.7%)	113 (0.7%)	82 (0.9%)	0.083
Gingival hemorrhage, *n* (%)	1,994 (7.5%)	1,214 (7.2%)	780 (8.2%)	0.004
Other hemorrhage, *n* (%)	295 (1.1%)	191 (1.1%)	104 (1.1%)	0.757
Vasogenic edema, *n* (%)	43 (0.2%)	29 (0.2%)	14 (0.1%)	0.627
No complication, *n* (%)	23,043 (87.0%)	14,826 (87.7%)	8,217 (85.9%)	<0.001

**Table 4:** Regression analyses evaluating the associated risk factors of intracerebral hemorrhage after thrombolysis.

**Index**	**Unadjusted**		**Adjusted**	
	**OR (95%CI)**	***P-*value**	**OR (95%CI)**	***P-*value**
Sex (female vs. male^*^)	1.287 (1.128–1.468)	<0.001	0.982 (0.856–1.126)	0.790
Age	1.037 (1.031–1.043)	<0.001	1.022 (1.015–1.028)	<0.001
Baseline NIHSS	1.062 (1.054–1.070)	<0.001	1.039 (1.031–1.048)	<0.001
DNT	1.004 (1.002–1.006)	<0.001	1.004 (1.002–1.006)	<0.001
Hospital levels(Tertiary vs. secondary^*^)	1.084 (0.928–1.268)	0.309	–	–
Arrival mode (Ambulance vs. other^*^)	1.766 (1.551–2.012)	<0.001	1.387 (1.211–1.587)	<0.001
Etiology (Cardioembolism vs. other^*^)	3.301 (2.864–3.806)	<0.001	2.155 (1.846–2.516)	<0.001

*CI, confidence interval; DNT, door to needle time; NIHSS, National Institute of Health stroke scale; OR, odds ratio*.

* * is the reference variable*.

In addition, the rate of patients with no complications was higher in male than in female patients (*P* < 0.05). The complications of gastrointestinal and other organ bleeding, as well as angioedema, were not different between male and female patients.

## Discussion

The current study showed that while sex was not associated with intracerebral hemorrhage or in-hospital mortality after adjusting for other predictors, the DNT was longer and the rates of intracerebral hemorrhage and in-hospital mortality were higher in female than in male patients.

At present, whether sex plays a role in treatment delays in patients with stroke remains controversial. Most studies revealed that female patients experience 20-min to 4-h delays in treatment compared with male patients (12–17); however, a few studies indicated no difference in treatment delays between male and female patients or short delays in female vs. male patients (8–11). In the current study, the ODT was not different between male and female patients, whereas the DNT was significantly longer in female vs. male patients. This finding demonstrates that in-hospital treatment delays were longer in female patients, but pre-hospital delays were not different between male and female patients. Some studies reported that atypical clinical manifestations, including headache, fatigue, disorientation, and mental and behavioral disorders, are common in female patients (18–20). These manifestations may be associated with delays in accurate diagnosis and treatment at the early stage. Due to the absence of information on clinical manifestations in our database, determining whether the longer DNT was related to the abovementioned factors in female patients was not possible. Notably, the median DNT was only 1 min longer in female than in male patients, which is obviously shorter than those reported by foreign studies. Furthermore, there may be merely limited clinical implication in the 1 min difference although it was statistically significant.

According to several established studies, female patients experience worse functional outcomes, higher mortality, and longer hospital stays than do male patients with stroke (8, 13, 21–27). A meta-analysis including 36 population-based and three randomized trials revealed that female sex is independently associated with post-stroke mortality (28). In addition, some studies also suggested that the age of onset and proportion of cardioembolism are higher in females, causing worse outcomes in female vs. male patients with stroke (20, 21, 27). In the current study, the mortality rate was higher in female than in male patients after thrombolysis. However, sex was not associated with an increased risk of in-hospital mortality after adjusting for covariates including age, baseline NIHSS scores, DNT, and TOAST classification. Therefore, worse outcomes in female patients are likely related to older age and confounding factors including cardioembolism. Our results generally agree with previous reports.

Our study particularly showed that the decrease in NIHSS score was greater at 7 days after thrombolysis in female than in male patients, although this was only marginally significant. According to various studies, female patients with stroke have conditions that are more severe before thrombolysis compared with male patients, and the difference in disease severity may be diminished by thrombolytic treatment (29–32). In this study, the NIHSS score was indeed higher in female patients than that in male patients before thrombolysis, which is consistent with established reports. However, only the NIHSS score before and after thrombolysis were recorded in our database, and this is far from reflecting functional outcomes. Therefore, we are not able to confirm the difference in functional outcome related to thrombolysis between female and male patients.

At present, only a few high-quality studies are available on differences in thrombolysis-related complications between male and female patients, and the results remain controversial. The Canadian Alteplase for Stroke Effectiveness Study, a multicenter registered trial, indicated that symptomatic intracerebral hemorrhage following thrombolysis occurs less in female than in male patients (32). Meanwhile, a meta-analysis involving 9,914 patients from 11 studies found no difference in thrombolysis-related complications between male and female patients (33). In our study, the rate of intracerebral hemorrhage after thrombolysis was higher in female patients, whereas the rate of patients with no complications was higher in male patients. However, sex was no longer associated with intracerebral hemorrhage after adjusting for age, disease severity, and relative pathogenic mechanism factors. Further studies with larger sample sizes performed across numerous centers are necessary to verify the findings of the present study.

The database used herein involves 902 hospitals from 29 provinces, covering most areas in China. To our knowledge, this registered study includes the largest sample size in China, implying strong generalizability and representativeness. Meanwhile, the study data were from the past 2 years, indicating the present study is a time-efficient evaluation of the potential problems in emergency treatments for stroke.

Our study is limited by several inherent shortcomings. First, the data in the current study were obtained retrospectively from an existing database. Only patients who were reported by each center and received thrombolysis were included in the database, whereas patients who did not receive thrombolytic treatment were not included. Therefore, we were not able to calculate the thrombolysis rate between male and female patients nor analyze the data in patients who did not receive thrombolysis. Nevertheless, such data are important for identifying new problems and improving the thrombolysis rate. Second, the database did not include information on patients' risk factors and previous histories, which made it impossible to analyze the effects of these factors on treatment and prognosis. In addition, only data on all-cause in-hospital mortality were available; thus, assessing the association between mortality and thrombolysis was difficult. The same situation existed in the analysis on the relationship between intracerebral hemorrhage and thrombolysis. Moreover, we cannot distinguishing symptomatic and asymptomatic ICH because some essential information were not recorded in the database. In fact the complications of symptomatic hemorrhage is more relevant to the prognosis, and a clear definition of symptomatic ICH should be adopted when relevant information is registered in the future, such as that in the European-Australian Cooperative Acute Stroke Study 2 (ECASS2) or a modified version of the Safe Implementation of Thrombolysis in Stroke Monitoring Study (mSITS-MOST) (34). Finally, data on modified Rankin scale scores, recurrent stroke, mortality in the long term, length of stay and duration of follow-up were not recorded, which are important factors for assessing thrombolysis outcomes. Consequently, the long-term prognosis after thrombolysis could not be determined in the study. These data should be collected in future similar studies.

## Conclusions

In-hospital treatment delays were longer in female than in male patients, and sex was not associated with increased in-hospital mortality after adjusting for other predictors. However, the rates of intracerebral hemorrhage and in-hospital mortality were higher in female patients. Furthermore, improvement in NIHSS score at 7 days after thrombolysis was greater in female than in male patients. This is the largest study to investigate the differences in intravenous thrombolysis between male and female patients with acute ischemic stroke in China. The obtained results are potentially useful for improving thrombolysis implementation and emergency treatment levels. Further studies are warranted to evaluate the long-term outcomes in the different sexes.

## Data Availability Statement

The raw data supporting the conclusions of this article will be made available by the authors, without undue reservation.

## Ethics Statement

The study protocol was approved by the ethics review committee of Peking Union Medical College Hospital of the Chinese Academy of Medical Sciences (review number: S-K988).

## Author Contributions

BP contributed conception and design of the study. BP, HL, and Z-qL organized the database. BC and S-dL performed the statistical analysis. BC wrote the first draft of the manuscript. All authors contributed to the article and approved the submitted version.

## Conflict of Interest

The authors declare that the research was conducted in the absence of any commercial or financial relationships that could be construed as a potential conflict of interest.
